# C—I⋯N short contacts as tools for the construction of the crystal packing in the crystal structure of 3,3′-(ethane-1,2-di­yl)bis­(6-iodo-3,4-di­hydro-2*H*-1,3-benzoxazine)

**DOI:** 10.1107/S2056989017005047

**Published:** 2017-04-07

**Authors:** Augusto Rivera, Jicli José Rojas, Jaime Ríos-Motta, Michael Bolte

**Affiliations:** a Universidad Nacional de Colombia, Sede Bogotá, Facultad de Ciencias, Departamento de Química, Cra 30 No. 45-03, Bogotá, Código Postal 111321, Colombia; b Institut für Anorganische Chemie, J. W. Goethe-Universität Frankfurt, Max-von Laue-Strasse 7, 60438 Frankfurt/Main, Germany

**Keywords:** crystal structure, short contacts, benzoxazines, phenolic resins

## Abstract

The asymmetric unit of the title compound, C_18_H_18_I_2_N_2_O_2_, consists of one half-mol­ecule, completed by the application of inversion symmetry. The mol­ecule adopts the typical structure for this class of bis-benxozazines, characterized by an *anti* orientation of the two benzoxazine rings around the central C—C bond. The oxazinic ring adopts a half-chair conformation. In the crystal, mol­ecules are linked by C—I⋯N short contacts [I⋯N = 3.378 (2) Å], generating layers lying parallel to the *bc* plane.

## Abstract 2

The packing of the title compound features short C—I⋯N contacts.

## Chemical context   

Benzoxazines have been studied for more than 70 years (Holly & Cope, 1944 ▸): they are heterocyclic compounds, which have the core structure of a benzene ring fused with an oxazine ring that can be readily synthesized by the Mannich reaction of mixing three components, either in solution or by a melt-state reaction using a combination of a phenolic derivative, formaldehyde, and a primary amine (Wattanathana *et al.*, 2014 ▸). The importance of these compounds is for the production of the corresponding polymers called polybenzoxazines, which have been developed as a class of ring-opening phenolic resins (Ishida & Sanders, 2000 ▸). However, the usefulness of benzoxazines as precursors for a class of thermosetting phenolic resins with excellent mechanical and thermal properties was not recognized until recently (Velez-Herrera & Ishida, 2009 ▸).
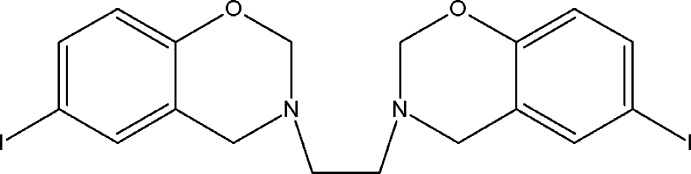


As the electrophilic character of the substituents affects the stability both of the reaction inter­mediates and the benzoxazine ring (Hamerton *et al.*, 2006 ▸), consequently, when *p*-iodo­phenol, formaldehyde and ethyl­enedi­amine were allowed to react in a molar ratio of 2:4:1, the title compound (I) was formed. This article forms part of our ongoing research into improving the understanding of the structural features resulting from replacement of the halogen substituent at the *para* position of the aromatic ring of bis-1,3-benzoxazines. So, an iodine functional bis-1,3-benzoxazine, namely 3,3′-(ethane-1,2-di­yl)bis­(6-iodo-3,4-di­hydro-2*H*-1,3-benzoxazine) has been synthesized in high yield and purity.

## Structural commentary   

**Figure 1:**
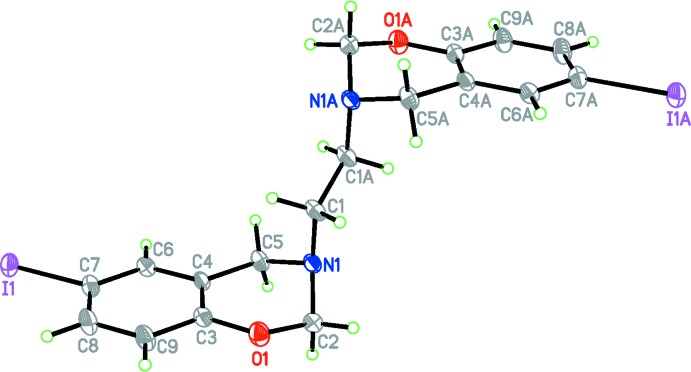
The mol­ecular structure of the title compound, with displacement ellipsoids drawn at the 50% probability level. Atoms labelled with the suffix A are generated using the symmetry operator (1 − *x*, 1 − *y*, 1 − *z*).

Similar to that observed in the crystal structure of the related compounds (Rivera *et al.*, 2010 ▸, 2016*a* ▸), the asymmetric unit of the title compound C_18_H_18_I_2_N_2_O_2_, contains one-half of the formula unit; a centre of inversion is located at the mid-point of the central C1—C1(1 − *x*, 1 − *y*, 1 − *z*) bond (see Fig. 1 ▸). The six-membered oxazine heterocyclic ring adopts a half-chair conformation, with puckering parameters *Q* = 0.482 (3) Å, θ =129.6 (2)°, φ = 283.6 (3)°: with respect to the plane formed by O1/C3/C4/C5, the deviations of C2 and N1 are 0.301 (3) and −0.320 (3) Å, respectively. The observed C—O bond length [1.376 (3) Å] is in a good agreement with the related *p*-fluoro and *p*-bromo structures (Rivera *et al.*, 2016*a* ▸,*b* ▸), but this value is shorter than for the the *p*-chloro derivative (Rivera *et al.*, 2010 ▸). The C7—I1 bond length [2.107 (3) Å] is in good agreement with the value reported for 4-iodo­phenol [2.104 (5) Å; Merz, 2006 ▸]. The C8—C9 bond length [1.378 (4) Å] is shorter than the average C–C bond length of benzene ring [1.398 (4) Å)]. The N1—C2 bond length [1.435 (3) Å] is significantly shorter than those of N1—C5 [1.474 (3) Å] and N1—C1 [1.478 (3) Å], probably due to the presence of a hyperconjugative inter­action between the lone-pair electrons of the nitro­gen atom and the anti­bonding *σ* orbital of C—O bond (*n*N→σ*_C2–O1_). Moreover, the C2—N1—C1 [112.6 (2)°] and C5—N1—C1 [113.0 (2)°] angles are larger than the mean value of *sp* ^3^ hybridization in ammonia (107°; Olovsson & Templeton, 1959 ▸).

## Supra­molecular features   

**Figure 2:**
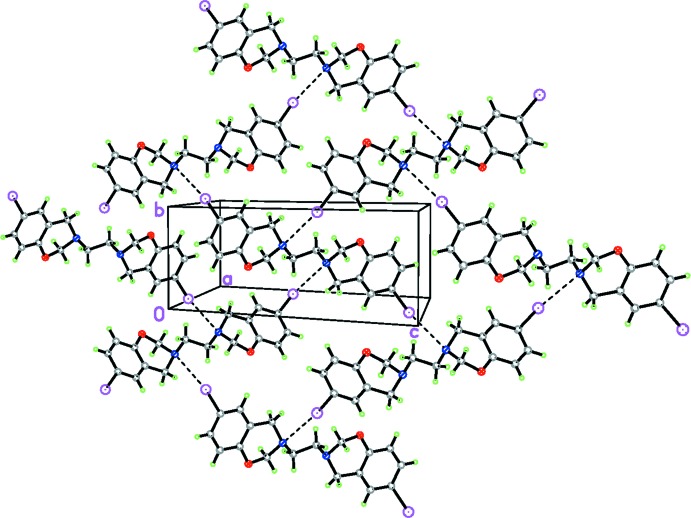
Crystal packing of (I), displaying C—I⋯N short contacts, which result in chains, forming layers propagating parallel to the *bc* plane.

The crystal-packing arrangement of the title compound is illustrated in Fig. 2 ▸. In contrast with related structures (Rivera *et al.*, 2016*a* ▸,*b* ▸, 2010 ▸), the absence of C—H⋯X or C—H⋯O inter­actions in the title compound is surprising. The packing of title compound is dominated by short contacts (Table 1 ▸), as indicated by a *PLATON* (Spek, 2009 ▸) analysis. Short C—I⋯N inter­actions (Table 1 ▸) are observed between neighboring mol­ecules; it is remarkable that these short contacts present in the crystal structure of (I) has structure-directing characteristics.

## Database survey   

A search of the Cambridge Structural Database (Groom *et al.*, 2016 ▸) for short N⋯I contacts between an N atom bonded to three C atoms and an I atom bonded to an aromatic ring yielded 47 entries with a distance of less than 3.5 Å. The search yielded four comparable structures, namely 3,3′-ethane-1,2-diylbis(6-methyl-3,4-di­hydro-2*H*-1,3-benzoxazine) (AXAKAM; Rivera *et al.*, 2011 ▸), 3,3′-ethyl­enebis(3,4-di­hydro-6-chloro-2*H*-1,3-benzoxazine), (NUQKAM; Rivera *et al.*, 2010 ▸), 3,3′-(ethane-1,2-di­yl)-bis­(6-meth­oxy-3,4-di­hydro-2*H*-1,3-benzoxazine) monohydrate (QEDDOU; Rivera *et al.*, 2012*b* ▸), 3,3′-ethane-1,2-diylbis(3,4-di­hydro-2*H*-1,3-benzoxazine) (SAGPUN; Rivera *et al.*, 2012*a* ▸).

## Synthesis and crystallization   

The title compound was prepared as described by Rivera *et al.* (1989 ▸). The reaction mixture was stored at room temperature for several weeks until a yellowish precipitate was formed. The solid was separated by filtration, washed with ethanol and crystallized from acetone solution. Yield 45.5%, m.p. 434 K.

## Refinement details   

Crystal data, data collection and structure refinement details are summarized in Table 2 ▸. All H atoms were located in the difference electron-density map. C-bound H atoms were fixed geometrically (C—H = 0.95 or 0.99Å) and refined using a riding-model approximation, with *U*_iso_(H) set to 1.2*U*_eq_ of the parent atom.

## Supplementary Material

Crystal structure: contains datablock(s) I. DOI: 10.1107/S2056989017005047/hb7668sup1.cif

Structure factors: contains datablock(s) I. DOI: 10.1107/S2056989017005047/hb7668Isup2.hkl

Supporting information file. DOI: 10.1107/S2056989017005047/hb7668Isup3.cml

CCDC reference: 1541561

Additional supporting information:  crystallographic information; 3D view; checkCIF report

## Figures and Tables

**Table 1:** Short-contact geometry (Å, °)

C—I	*X*	C—I	I···*X*	C—I···*X*
C7—I1	N1^i^	2.107 (3)	3.378 (2)	169.13 (9)

Symmetry code: (i) *x*, −*y*, 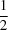 + *z*.

**Table 2:** Experimental details

Crystal data	
Chemical formula	C_18_H_18_I_2_N_2_O_2_
*M*_r_	548.14
Crystal system, space group	Monoclinic, *C*2/*c*
Temperature (K)	173
*a*, *b*, *c* (Å)	20.4200 (9), 5.9477 (2), 17.8414 (8)
β (°)	123.607 (3)
*V* (Å^3^)	1804.69 (14)
*Z*	4
Radiation type	Mo *K*α
μ (mm^−1^)	3.50
Crystal size (mm)	0.29 × 0.27 × 0.27

Data collection	
Diffractometer	Stoe IPDS II two-circle
Absorption correction	Multi-scan (*X-AREA*; Stoe & Cie, 2001 ▸)
*T*_min_, *T*_max_	0.395, 1.000
No. of measured, independent and observed [*I* > 2σ(*I*)] reflections	39259, 2531, 2456
*R*_int_	0.076
(sin θ/λ)_max_ (Å^−1^)	0.697

Refinement	
*R*[*F* ^2^ > 2σ(*F* ^2^)], *wR*(*F* ^2^), *S*	0.030, 0.076, 1.22
No. of reflections	2531
No. of parameters	110
H-atom treatment	H-atom parameters constrained
Δρ_max_, Δρ_min_ (e Å^−3^)	1.46, −1.35

Computer programs: *X-AREA* (Stoe & Cie, 2001 ▸), *SHELXS97*, *SHELXL97* and *XP* in *SHELXTL-Plus* (Sheldrick, 2008 ▸), *SHELXL2016* (Sheldrick, 2015 ▸).

## supplementary crystallographic information

### Crystal data

**Table:**

C_18_H_18_I_2_N_2_O_2_	*F*(000) = 1048
*M**_r_* = 548.14	*D*_x_ = 2.017 Mg m^−^^3^
Monoclinic, *C*2/*c*	Mo *K*α radiation, λ = 0.71073 Å
*a* = 20.4200 (9) Å	Cell parameters from 39259 reflections
*b* = 5.9477 (2) Å	θ = 3.3–29.9°
*c* = 17.8414 (8) Å	µ = 3.50 mm^−^^1^
β = 123.607 (3)°	*T* = 173 K
*V* = 1804.69 (14) Å^3^	Block, colourless
*Z* = 4	0.29 × 0.27 × 0.27 mm

### Data collection

**Table:**

Stoe IPDS II two-circle diffractometer	2456 reflections with *I* > 2σ(*I*)
Radiation source: Genix 3D IµS microfocus X-ray source	*R*_int_ = 0.076
ω scans	θ_max_ = 29.7°, θ_min_ = 3.6°
Absorption correction: multi-scan (X-AREA; Stoe & Cie, 2001)	*h* = −28→28
*T*_min_ = 0.395, *T*_max_ = 1.000	*k* = −7→8
39259 measured reflections	*l* = −24→24
2531 independent reflections	

### Refinement

**Table:**

Refinement on *F*^2^	Hydrogen site location: inferred from neighbouring sites
Least-squares matrix: full	H-atom parameters constrained
*R*[*F*^2^ > 2σ(*F*^2^)] = 0.030	*w* = 1/[σ^2^(*F*_o_^2^) + (0.0378*P*)^2^ + 4.0804*P*] where *P* = (*F*_o_^2^ + 2*F*_c_^2^)/3
*wR*(*F*^2^) = 0.076	(Δ/σ)_max_ = 0.002
*S* = 1.22	Δρ_max_ = 1.46 e Å^−^^3^
2531 reflections	Δρ_min_ = −1.35 e Å^−^^3^
110 parameters	Extinction correction: SHELXL2016 (Sheldrick, 2015), Fc^*^=kFc[1+0.001xFc^2^λ^3^/sin(2θ)]^-1/4^
0 restraints	Extinction coefficient: 0.0034 (2)

### Special details

**Table:**

Geometry. All esds (except the esd in the dihedral angle between two l.s. planes) are estimated using the full covariance matrix. The cell esds are taken into account individually in the estimation of esds in distances, angles and torsion angles; correlations between esds in cell parameters are only used when they are defined by crystal symmetry. An approximate (isotropic) treatment of cell esds is used for estimating esds involving l.s. planes.

### Fractional atomic coordinates and isotropic or equivalent isotropic displacement parameters (Å^2^)

**Table:**

	*x*	*y*	*z*	*U*_iso_*/*U*_eq_	
I1	0.60686 (2)	−0.05887 (3)	0.93105 (2)	0.02375 (9)	
O1	0.68205 (12)	0.6146 (3)	0.71878 (13)	0.0231 (4)	
N1	0.60465 (12)	0.4020 (4)	0.58050 (14)	0.0174 (4)	
C1	0.53347 (15)	0.5410 (4)	0.54603 (16)	0.0207 (5)	
H1A	0.545662	0.699539	0.541455	0.025*	
H1B	0.516975	0.534394	0.588747	0.025*	
C2	0.67584 (15)	0.5254 (5)	0.63881 (17)	0.0214 (5)	
H2A	0.678708	0.651795	0.604633	0.026*	
H2B	0.721287	0.425521	0.657944	0.026*	
C3	0.66459 (15)	0.4585 (4)	0.76215 (17)	0.0187 (4)	
C4	0.62686 (14)	0.2547 (4)	0.72243 (15)	0.0171 (4)	
C5	0.60538 (15)	0.1994 (4)	0.62851 (16)	0.0199 (4)	
H5A	0.643793	0.090235	0.632478	0.024*	
H5B	0.552776	0.128136	0.594033	0.024*	
C6	0.61047 (15)	0.1080 (4)	0.77133 (16)	0.0191 (4)	
H6	0.585192	−0.031336	0.745502	0.023*	
C7	0.63083 (15)	0.1641 (5)	0.85739 (16)	0.0208 (5)	
C8	0.66757 (17)	0.3687 (5)	0.89574 (17)	0.0250 (5)	
H8	0.681043	0.407706	0.954297	0.030*	
C9	0.68424 (17)	0.5143 (5)	0.84831 (17)	0.0236 (5)	
H9	0.709313	0.653654	0.874439	0.028*	

### Atomic displacement parameters (Å^2^)

**Table:**

	*U*^11^	*U*^22^	*U*^33^	*U*^12^	*U*^13^	*U*^23^
I1	0.02726 (12)	0.02815 (12)	0.01965 (12)	0.00072 (6)	0.01537 (9)	0.00344 (6)
O1	0.0281 (9)	0.0214 (8)	0.0185 (8)	−0.0058 (7)	0.0121 (7)	−0.0023 (7)
N1	0.0167 (9)	0.0205 (9)	0.0129 (8)	0.0030 (7)	0.0069 (7)	0.0024 (7)
C1	0.0207 (11)	0.0228 (11)	0.0130 (10)	0.0046 (9)	0.0060 (9)	0.0010 (8)
C2	0.0188 (10)	0.0271 (12)	0.0168 (10)	−0.0008 (9)	0.0089 (9)	0.0013 (9)
C3	0.0185 (10)	0.0207 (11)	0.0150 (10)	0.0001 (8)	0.0081 (9)	0.0021 (8)
C4	0.0166 (9)	0.0219 (10)	0.0109 (9)	0.0022 (8)	0.0064 (8)	0.0017 (8)
C5	0.0248 (11)	0.0198 (10)	0.0133 (9)	0.0014 (9)	0.0093 (9)	−0.0005 (8)
C6	0.0196 (10)	0.0197 (10)	0.0154 (10)	0.0005 (8)	0.0081 (8)	0.0009 (8)
C7	0.0235 (11)	0.0227 (11)	0.0163 (10)	0.0011 (9)	0.0110 (9)	0.0030 (9)
C8	0.0338 (13)	0.0266 (13)	0.0156 (10)	−0.0017 (11)	0.0144 (10)	−0.0019 (9)
C9	0.0311 (13)	0.0224 (11)	0.0151 (10)	−0.0037 (10)	0.0114 (10)	−0.0032 (9)

### Geometric parameters (Å, º)

**Table:**

I1—C7	2.107 (3)	C3—C4	1.400 (3)
O1—C3	1.376 (3)	C4—C6	1.399 (3)
O1—C2	1.460 (3)	C4—C5	1.515 (3)
N1—C2	1.435 (3)	C5—H5A	0.9900
N1—C5	1.474 (3)	C5—H5B	0.9900
N1—C1	1.478 (3)	C6—C7	1.391 (3)
C1—C1^i^	1.523 (5)	C6—H6	0.9500
C1—H1A	0.9900	C7—C8	1.394 (4)
C1—H1B	0.9900	C8—C9	1.378 (4)
C2—H2A	0.9900	C8—H8	0.9500
C2—H2B	0.9900	C9—H9	0.9500
C3—C9	1.397 (4)		
			
I1···N1^ii^	3.378 (2)		
			
C3—O1—C2	113.3 (2)	C6—C4—C5	122.1 (2)
C2—N1—C5	108.45 (19)	C3—C4—C5	119.3 (2)
C2—N1—C1	112.6 (2)	N1—C5—C4	111.6 (2)
C5—N1—C1	113.0 (2)	N1—C5—H5A	109.3
N1—C1—C1^i^	111.0 (3)	C4—C5—H5A	109.3
N1—C1—H1A	109.4	N1—C5—H5B	109.3
C1^i^—C1—H1A	109.4	C4—C5—H5B	109.3
N1—C1—H1B	109.4	H5A—C5—H5B	108.0
C1^i^—C1—H1B	109.4	C7—C6—C4	120.7 (2)
H1A—C1—H1B	108.0	C7—C6—H6	119.7
N1—C2—O1	113.5 (2)	C4—C6—H6	119.7
N1—C2—H2A	108.9	C6—C7—C8	120.1 (2)
O1—C2—H2A	108.9	C6—C7—I1	120.46 (19)
N1—C2—H2B	108.9	C8—C7—I1	119.41 (18)
O1—C2—H2B	108.9	C9—C8—C7	119.7 (2)
H2A—C2—H2B	107.7	C9—C8—H8	120.2
O1—C3—C9	116.9 (2)	C7—C8—H8	120.2
O1—C3—C4	122.7 (2)	C8—C9—C3	120.5 (3)
C9—C3—C4	120.3 (2)	C8—C9—H9	119.7
C6—C4—C3	118.6 (2)	C3—C9—H9	119.7
			
C2—N1—C1—C1^i^	150.8 (3)	C1—N1—C5—C4	−77.2 (2)
C5—N1—C1—C1^i^	−85.9 (3)	C6—C4—C5—N1	161.7 (2)
C5—N1—C2—O1	−65.2 (3)	C3—C4—C5—N1	−18.6 (3)
C1—N1—C2—O1	60.5 (3)	C3—C4—C6—C7	0.4 (4)
C3—O1—C2—N1	47.5 (3)	C5—C4—C6—C7	−179.9 (2)
C2—O1—C3—C9	167.3 (2)	C4—C6—C7—C8	0.3 (4)
C2—O1—C3—C4	−14.5 (3)	C4—C6—C7—I1	−179.47 (18)
O1—C3—C4—C6	−179.0 (2)	C6—C7—C8—C9	−0.6 (4)
C9—C3—C4—C6	−0.9 (4)	I1—C7—C8—C9	179.2 (2)
O1—C3—C4—C5	1.2 (4)	C7—C8—C9—C3	0.1 (4)
C9—C3—C4—C5	179.3 (2)	O1—C3—C9—C8	178.9 (3)
C2—N1—C5—C4	48.3 (3)	C4—C3—C9—C8	0.7 (4)

Symmetry codes: (i) −*x*+1, −*y*+1, −*z*+1; (ii) *x*, −*y*, *z*+1/2.

## References

[bb1] Groom, C. R., Bruno, I. J., Lightfoot, M. P. & Ward, S. C. (2016). *Acta Cryst.* B**72**, 171–179. 10.1107/S2052520616003954 PMC4822653 27048719

[bb2] Hamerton, I., Howlin, B. J. & Mitchell, A. L. (2006). *React. Funct. Polym.* **66**, 21–39.

[bb3] Holly, F. W. & Cope, A. C. (1944). *J. Am. Chem. Soc.* **66**, 1875–1879.

[bb4] Ishida, H. & Sanders, D. P. (2000). *J. Polym. Sci. Part B*, **38**, 3289–3301.

[bb5] Merz, K. (2006). *Cryst. Growth Des.* **6**, 1615–1619.

[bb6] Olovsson, I. & Templeton, D. H. (1959). *Acta Cryst.* **12**, 832–836.

[bb7] Rivera, A., Aguilar, Z., Clavijo, D. & Joseph-Nathan, P. (1989). *An. Quim. Ser. C*, **85**, 9–10.

[bb8] Rivera, A., Camacho, J., Ríos-Motta, J., Fejfarová, K. & Dušek, M. (2012*a*). *Acta Cryst.* E**68**, o148. 10.1107/S1600536811053530 PMC3254492 22259434

[bb9] Rivera, A., Camacho, J., Ríos-Motta, J., Kučeraková, M. & Dušek, M. (2012*b*). *Acta Cryst.* E**68**, o2734. 10.1107/S1600536812035519 PMC3435746 22969617

[bb10] Rivera, A., Camacho, J., Ríos-Motta, J., Pojarová, M. & Dušek, M. (2011). *Acta Cryst.* E**67**, o2028. 10.1107/S1600536811027139 PMC3213478 22091057

[bb11] Rivera, A., Rojas, J. J., Ríos-Motta, J. & Bolte, M. (2016*a*). *Acta Cryst.* E**72**, 1645–1647. 10.1107/S2056989016016509 PMC5095852 27840727

[bb12] Rivera, A., Rojas, J. J., Ríos-Motta, J. & Bolte, M. (2016*b*). *Acta Cryst.* E**72**, 1509–1511. 10.1107/S2056989016015243 PMC5050787 27746952

[bb13] Rivera, A., Rojas, J. J., Ríos-Motta, J., Dušek, M. & Fejfarová, K. (2010). *Acta Cryst.* E**66**, o1134. 10.1107/S1600536810014248 PMC2979041 21579183

[bb14] Sheldrick, G. M. (2008). *Acta Cryst.* A**64**, 112–122. 10.1107/S0108767307043930 18156677

[bb15] Sheldrick, G. M. (2015). *Acta Cryst.* C**71**, 3–8.

[bb16] Spek, A. L. (2009). *Acta Cryst.* D**65**, 148–155. 10.1107/S090744490804362X PMC2631630 19171970

[bb17] Stoe & Cie (2001). *X-AREA* and *X-RED32*. Stoe & Cie, Darmstadt, Germany.

[bb18] Velez-Herrera, P. & Ishida, H. (2009). *J. Fluor. Chem.* **130**, 573–580.

[bb19] Wattanathana, W., Nonthaglin, S., Veranitisagul, C., Koonsaeng, N. & Laobuthee, A. J. (2014). *J. Mol. Struct.* **1074**, 118–125.

